# Advancements in immunotherapy for colorectal cancer treatment: a comprehensive review of strategies, challenges, and future prospective

**DOI:** 10.1007/s00384-024-04790-w

**Published:** 2024-12-28

**Authors:** Vaishak Kaviyarasan, Alakesh Das, Dikshita Deka, Biki Saha, Antara Banerjee, Neeta Raj Sharma, Asim K. Duttaroy, Surajit Pathak

**Affiliations:** 1 Faculty of Allied Health Sciences, Chettinad Hospital and Research Institute (CHRI), Chettinad Academy of Research and Education (CARE), Kelambakkam, Chennai, Tamil Nadu 603103 India; 2 School of Bioengineering and Biosciences, Lovely Professional University, Phagwara, Punjab India; 3 Department of Nutrition, Institute of Basic Medical Sciences, Faculty of Medicine, University of Oslo, Oslo, Norway

**Keywords:** Colorectal cancer, Immune system, Immunotherapy, Immune checkpoint inhibitors, Therapeutics, Diagnosis

## Abstract

**Purpose:**

Colorectal cancer (CRC) remains one of the leading causes of cancer-related mortality worldwide. Metastatic colorectal cancer (mCRC) continues to present significant challenges, particularly in patients with proficient mismatch repair/microsatellite stable (pMMR/MSS) tumors. This narrative review aims to provide recent developments in immunotherapy for CRC treatment, focusing on its efficacy and challenges.

**Methods:**

This review discussed the various immunotherapeutic strategies for CRC treatment, including immune checkpoint inhibitors (ICIs) targeting PD-1 and PD-L1, combination therapies involving ICIs with other modalities, chimeric antigen receptor T-cell (CAR-T) cell therapy, and cancer vaccines. The role of the tumor microenvironment and immune evasion mechanisms was also explored to understand their impact on the effectiveness of these therapies.

**Results:**

This review provides a comprehensive update of recent advancements in immunotherapy for CRC, highlighting the potential of various immunotherapeutic approaches, including immune checkpoint inhibitors, combination therapies, CAR-T therapy, and vaccination strategies. The results of checkpoint inhibitors, particularly in patients with MSI-H/dMMR tumors, which have significant improvements in survival rates have been observed. Furthermore, this review also addresses the challenges faced in treating pMMR/MSS CRC, which remains resistant to immunotherapy.

**Conclusion:**

Immunotherapy plays a significant role in the treatment of CRC, particularly in patients with MSI-H/dMMR tumors. However, many challenges remain, especially in treating pMMR/MSS CRC. This review discussed the need for further research into combination therapies, biomarker development, CAR-T cell therapy, and a deeper understanding of immune evasion mechanisms for CRC treatment.

## Introduction

Colorectal cancer (CRC) is one of the most prevalent forms of cancer and cancer-related death [1]. Although adults aged 50 and older account for most CRC cases, 12% of the cases are diagnosed in people under 50. Most CRC cases are sporadic, and only a few are hereditary [1, 2]. Family history, a high red and processed meat diet, inflammatory bowel disease, and obesity are the major risk factors for CRC [1–3]. Optimizing surgical resection for individuals with localized disease has significantly improved survival rates of 5 to 10 years [2, 3]. A significant number of patients are diagnosed with metastatic colorectal cancer (mCRC), and the prognosis for individuals with distant metastases is generally poor [1, 2]. Even though surgery successfully removes the visible tumor from CRC patients with localized disease, however, they experience a relapse due to micro-metastases present at the time of surgery [1–4]. Patients with oligometastatic disease who receive systemic therapy and tumor excision have a higher survival rate compared to those with mCRC [5, 6]. Despite the elevated benefits of targeted therapy and chemotherapy, it is important to develop new and efficient therapeutic approaches promptly to enhance the overall survival rates of patients with mCRC [7]. Immunotherapy has emerged as the most promising paradigm shift in cancer treatment [7, 8]. The primary goal of immunotherapy is to stimulate the immune response to inhibit tumor development. Immunotherapy that promotes and supports appropriate immunological conditions in CRC patients can increase patient’s life expectancy [6, 7].

Following early successes in the treatment of melanoma, immunotherapy has become a popular therapeutic strategy for several solid tumors, including CRC. Pembrolizumab and nivolumab, both antibodies targeting the PD-1 protein, have effectively treated mCRC in patients with deficient mismatch repair (dMMR) or high microsatellite instability (MSI-H) [7, 8]. The primary challenge is to develop effective immunotherapeutic strategies for proficient mismatch repair (pMMR) or microsatellite stable (MSS) cancers, which account for 95% of mCRC cases [9]. Recent research is investigating combination therapies that integrate immunotherapy with chemotherapy, targeted therapy, and radiation to improve efficacy in pMMR/MSS CRC [10]. The tumor microenvironment (TME) significantly influences immune evasion, and strategies to modify the TME are being explored to enhance immunotherapy outcomes [11]. In contrast to dMMR/MSI-H, tumors with the pMMR/MSS phenotype frequently show reduced tumor mutation loads and fewer tumor-infiltrating lymphocytes, which leads to immune tolerance and evasion within the TME [9, 11]. Gaining a comprehensive understanding of the immune system’s complex interactions and diversity within the TME, can aid in identifying predictive biomarkers and developing new therapeutic strategies to enhance anti-tumor immunity in patients with mCRC [12].

This review offers an in-depth analysis and discusses most of the updated information in a single article from the available research findings on the dynamic role of immunotherapy in managing CRC. Through a comprehensive analysis of recent developments, challenges, and potential integration with established therapies, this review provides valuable perspectives on the potential efficacy of immunotherapy in treating CRC and improving the quality of life for individuals diagnosed with CRC.

## Immune system and cancer

Cancer cells can evolve immune-evasion pathways to grow and spread in an uncontrolled manner [13]. Effector lymphocytes, particularly CD8 + T cells, are essential for antitumor responses but often become exhausted in the TME due to chronic stimulation and adverse conditions like high reactive oxygen species (ROS). Effective immunotherapies, such as Programmed Cell Death Protein 1 (PD1) and Programmed Death-Ligand 1 (PDL1) blockade, stimulate peripheral immune responses, driving new T cell clones into the TME, which are essential for overcoming local immune dysfunction [13, 14]. Systemic immune biomarkers, including specific cytokine levels and immune cell ratios, can predict responses to immunotherapy, highlighting the importance of peripheral immune health [14]. Tumor burden impairs systemic immunity, weakening responses to secondary challenges, but interventions like tumor resection and immune-stimulating agents can restore the function. Advanced single-cell technologies and mechanistic studies are essential for mapping the immune systems and designing therapies to restore a healthy immune state, which is crucial for effective cancer treatment [15].

### Immune surveillance

The immune system identifies cancer cells by detecting antigens on their surface. Tumor cells can evade this detection through various mechanisms, including downregulating antigens, activating immune checkpoints like PD-L1, and making an immunosuppressive environment [16–18]. These strategies help tumors escape immune destruction. In CRC, tumor cells suppress antigen production, making it harder for immune cells to recognize them, and they recruit immune-suppressive cells to inhibit immune responses against them [16–21].

### Immune evasion by cancer cells

Immune evasion refers to the mechanism for avoiding the immune system’s recognition and elimination of tumors. Cancer cells possess the ability to adapt and grow within the complex environment of the human body [21, 22]. However, cancer cells can manipulate immune checkpoints to reduce immune responses and create an immunosuppressive environment that hinders immune cell activity to avoid this surveillance [22, 23]. Cancer cells can activate immune checkpoint molecules like PD-L1, which send inhibitory signals to immune cells, reducing their response [24]. These PD-L1 are generally expressed by macrophages, some activated T cells, B cells, DC, and some epithelial cells, especially under inflammatory conditions [25]. Tumor cells express PD-L1 as an adaptive immune mechanism to evade antitumor responses [26]. It has been shown that IFN-γ causes PD-L1 upregulation in ovarian cancer cells, associated with disease progression [25, 26].

Inhibiting the IFN-γ receptor 1 may lower PD-L1 expression in acute myeloid leukemia mouse models via the MEK/ERK and MYD88/TRAF6 pathways [27]. IFN-γ activates protein kinase D isoform 2 (PKD2), essential for regulating PD-L1. Inhibiting PKD2 activity reduces PD-L1 expression and enhances the antitumor immune response [28]. Immune evasion is also facilitated by modifications in the antigen presentation and the generation of immunosuppressive substances. Cancer cells also inhibit or modify the surface antigens, which makes it challenging for immune cells to identify them as abnormal [29]. Additionally, immunosuppressive substances like IL-10 and TGF-β can be generated by cancer cells, which suppresses immune cell activity and develops an immunosuppressive microenvironment around the tumor [30].

### Immune response against cancer

Cytotoxic T cells kill cancer cells by releasing cytotoxic molecules, like perforin and granzymes, that triggers cell death [31]. Also, immune cells can secrete cytokines, including interferons and interleukins, which boost immune cell activity and recruit other immune cells to the tumor site [32]. Activated T cells, particularly cytotoxic T cells, target and destroy cancer cells by releasing cytotoxic molecules and cytokines [33]. However, cancer cells can exploit immune checkpoints to evade this response. Immunotherapies, such as checkpoint inhibitors and adoptive T cell therapy, aim to counter these evasion mechanisms and enhance the immune system response against CRC [34].

The immune system, especially T lymphocytes, detects specific tumor antigens generated on CRC cells as abnormal cells. As a result of this identification, T cells get activated, multiply, and differentiate into effector cells like neutrophils, basophils, and eosinophils [35]. Additionally, new drugs (monalizumab, lirilumab) that target immunosuppressive cells within tumors are improving the effectiveness of checkpoint inhibitors, thus enhancing overall immunotherapy. These advancements offer new hope for cancer treatments [36–38].

### Immune resistance mechanisms specific to CRC

Understanding the CRC-specific immune resistance mechanisms is essential for developing innovative therapies to target and overcome immune resistance mechanisms in CRC. Commonly, counteract antigen alterations, block immune checkpoints, disrupt immunosuppressive signals, and modulate the TME, researchers aim to improve the effectiveness of immunotherapy and ultimately enhance treatment outcomes for patients with CRC [39].

#### Deficiency in tumor antigen generation and presentation

Spontaneous T and B cell immunity against tumor antigens indicates that cytotoxic innate and adaptive immune cells can regulate tumor development [40]. However, as tumors progress, cancer cells develop pathways similar to peripheral immune tolerance to evade immune attacks [40]. It is done by avoiding recognition of tumor antigens and inhibiting the immune response. Cancer cells involve this by losing or downregulating MHC class I (major histocompatibility complex class I) molecules, which are crucial for cell-mediated immunity. Understanding these mechanisms is essential for improving immunotherapy strategies [41]. Elucidating the mechanisms responsible for the deficiency in tumor antigen presentation is crucial in developing strategies to enhance the immune recognition of cancer cells and improve the effectiveness of immunotherapies in treating CRC patients [42].

##### MHC downregulation and antigen presentation defects

Defects in antigen processing inside cancer cells can produce insufficient tumor antigens that MHC molecules can bind to and present [43]. Because of this, immune cells, especially cytotoxic T cells, may fail to recognize and efficiently target cancer cells, allowing them to evade immune surveillance [43, 44]. Further, MHC-I is downregulated in 40–90% of human tumors, frequently indicating a poor prognosis. Loss of MHC-I expression, often in cancer cells, contributes to tumor immune evasion [44]. These findings imply that MHC molecules may function directly as tumor suppressors to regulate tumor survival and development. Future studies are required on the recovery of MHC-I expression in tumor cells from various histological origins, investigating its impact on immune recognition and the intrinsically cancerous properties of tumor cells, which are the areas of importance [40–44].

##### Low tumor mutational burden and neoantigens

Colorectal tumors with a low tumor mutational burden (TMB) have fewer genetic alterations, translating to a limited pool of neoantigens, antigens generated from tumor-specific mutations [45]. Additionally, it was revealed that despite low TMB, the tumors from every patient with MSS CRC show clonal expected neoantigens [46, 47]. In MSI CRC, these neoantigens are generally expressed at lower levels. Similarly, it was shown that this low expression hinders effective cross-priming and accelerates T cell dysfunction [47]. Low TMB does not always mean no neoantigens, although it can limit the pool. In low TMB tumors, the immune system can detect neoantigens despite lower expression [47, 48].

#### Immune suppression in the tumor microenvironment

In CRC, the TME may be preventing appropriate antigen presentation and immune activation. Myeloid-derived suppressor cells (MDSC), regulatory T cells (Tregs), and cytokines like TGF-β may all work together to produce an immunosuppressive environment that inhibits the immune system’s response [49]. Numerous lines of evidence point to an essential function for immune monitoring in controlling CRC-related tumor progression [50]. As the CRC microenvironment evolves, it increasingly suppresses the immune response triggered by tumor invasion, allowing tumor cells to evade immune detection. Understanding the immunosuppressive mechanisms in CRC is essential for developing effective immunotherapeutic strategies in the future [51].

#### The roles and functions of Wnt and MAPK signaling pathways in immune evasion

In CRC, the WNT signaling pathway has two functions: it promotes immune evasion and tumor growth [52]. Canonical Wnt signaling is hyperactivated in many human CRCs due to genetic alterations of the negative Wnt regulator *APC* [53]. The MAPK pathway contributes to immune evasion by governing cytokine production, impairing immune cell functionality, encouraging immune checkpoint expression, and aiding tumor-associated angiogenesis [53, 54]. Several solid tumors, including CRC, have been related to the MAPK pathway, recognized as an oncogenic driver [54]. The dysregulated epidermal growth factor receptor (EGFR)/MAPK signaling pathway plays an oncogenic role in the initiation and development of CRC. Targeting MAPK disrupted the development of cultured CRC cells, occasionally causing them to shift toward an undesirable stem cell-like state [52–54].

Recent advances have underscored the crucial roles of Wnt and MAPK signaling pathways in cancer progression and immune evasion [55]. In CRC, dysregulated Wnt signaling often leads to immune suppression by driving tumor-associated macrophages (TAMs) toward an M2 phenotype [55, 56]. Strategies such as Wnt component inhibitors and novel drug delivery systems are enhancing the bioavailability and efficacy of these therapies [56]. Meanwhile, the MAPK pathway, although less extensively studied, plays a significant role in immune regulation and interacts with Wnt signaling to influence immune cell behavior and contribute to therapy resistance [55–57]. Combining therapies that target both Wnt and MAPK pathways with conventional treatments and immune checkpoint inhibitors (ICIs) presents a promising approach to overcoming cancer’s immune evasion mechanisms and enhancing patient outcomes [57].

#### Immune response specific to the right and left colon

CRC is one of the most frequent cancers worldwide, with differences in incidence, survival rates, and molecular features between right-sided (RCRC) and left-sided (LCRC). Sessile serrated or mucinous adenocarcinomas with flat shape, MSI-high, and peritoneal metastasis are common in RCRC, which originates from the cecum and ascending colon. RCRC had greater early-stage survival than LCRC, but worse advanced-stage results. Tumors often have MLH1 and MSH2 mutations, making this cancer more common in older persons and women [58]. In contrast, LCRC originates from the descending and sigmoid colon and manifests as tubular or villous adenocarcinomas with polypoid morphology, CIN-high chromosomal instability, and a higher risk of liver or lung metastasis. LCRC tumors often have *APC*, *KRAS*, and *TP53* mutations. Anti-VEGF medicines improve RCRC patient’s outcomes, but traditional chemotherapies worsen them. LCRC patients benefit better from anti-EGFR therapy. Anti-CTLA-4 treatments have shown minimal success in treating MSI-high RCRC tumors, whereas PD-1 inhibitors like pembrolizumab and nivolumab have demonstrated significant efficacy, leading to improved responses and prolonged survival in patients with these tumors due to their ability to block PD-1 and enhance the immune system’s ability to recognize and attack cancer cells [59]. Despite molecular differences, right-sided and left-sided colon tumors have distinct immune cell distribution and activity, including mucosal-associated invariant T (MAIT) and γδ T cells. RCRC tumors contain more tumor-infiltrating MAIT cells and higher serum carcinoembryonic antigen (CEA) levels. However, MAIT cells have decreased IFN-γ production, a key cytokine for antitumor immunity, and altered cytokine secretion patterns, including increased IL-17 [60]. Right-sided malignancies have worse prognoses and tend to spread to the abdomen.

In contrast, left-sided tumors often spread to the lungs and liver. Different immune profiles and responses to chemotherapy, including immune modulation medications like IL-17 and oxaliplatin, imply tumor location may affect growth and success of the treatment strategies. Some studies show greater 5-year disease-free survival for early-stage right-sided malignancies, but others show increased mortality and worse prognoses. More research is needed to understand these distinctions and create tumor-sided therapies [61].

## Immunological modulations associated with CRC

The immune cells that penetrate the TME are NK cells, dendritic cells, T cells, and macrophages, which can respond to cancer cells to release a variety of cytokines, chemokines, and growth factors that can promote or inhibit tumor growth [62]. The prognosis and survival of CRC patients are enhanced when there is a high infiltration of memory T cells and cytotoxic T lymphocytes (CTL) to the tumor site [63]. Tumor-infiltrating T cells (TIL) can be used as a prognostic indicator for CRC using immunohistochemical staining to determine their density and functional state [64]. Immune checkpoint molecules govern immune responses by establishing a proper equilibrium among activating and inhibiting signals of immune cells [63, 64]. PD-1 and its ligand, PD-L1 and CTLA-4, are examples of immunological checkpoint molecules. Immunological checkpoint medications targeting these inhibitory pathways have exhibited promising results in managing CRC by stimulating anti-tumor immune responses [19]. Tumor-associated antigens (TAA) are proteins that might trigger an immune response and are generated by cancer cells. TAA in CRC includes MUC1 and CEA. Cancer vaccines and immune-based treatments can target TAA to elicit an immune response against tumor cells [65].

## Immunotherapeutic approach to CRC

**Fig. 1:**
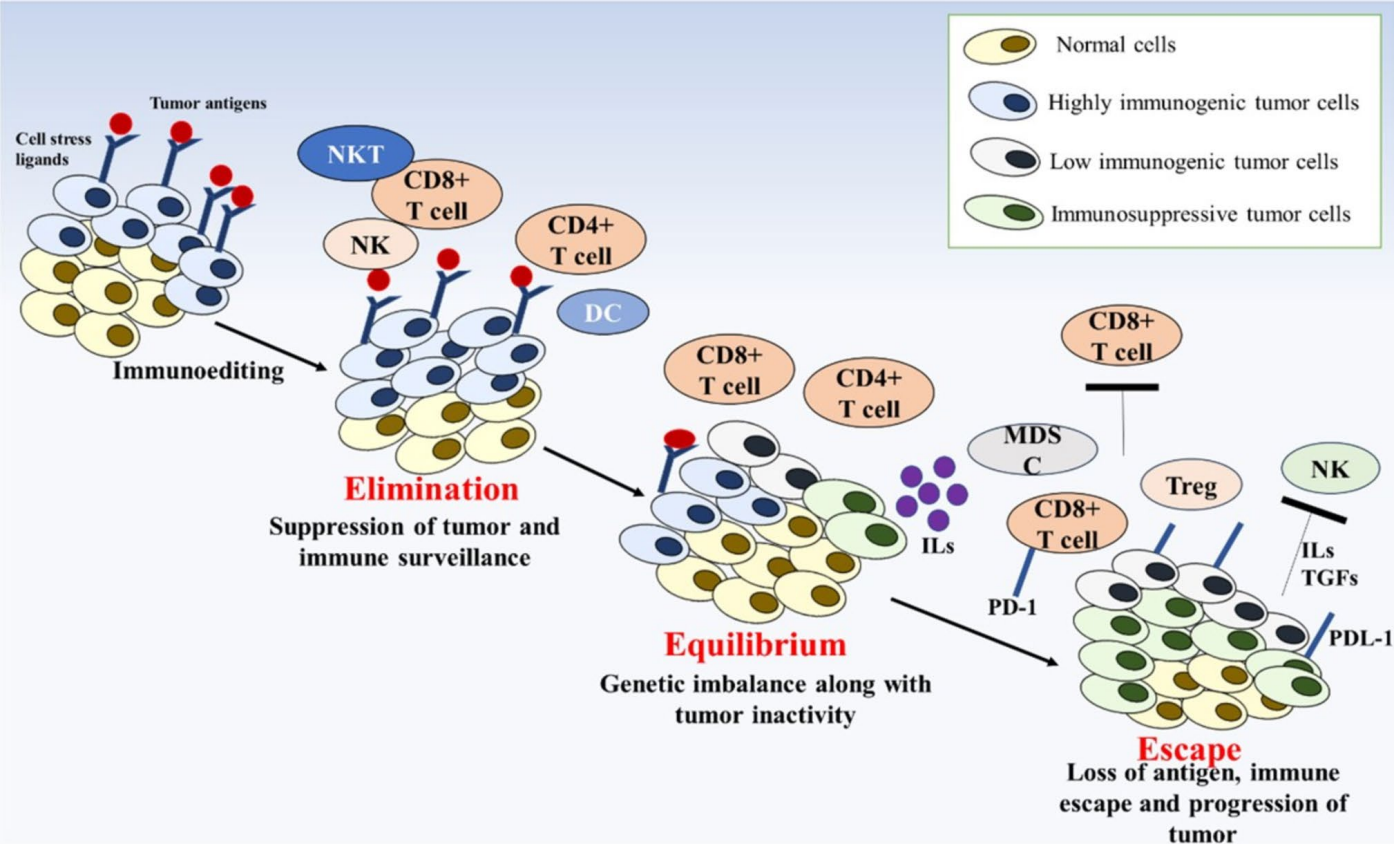
Schematic diagram representing the stages associated with the cancer immunoediting mechanism

The use of immunotherapy in managing CRC is still being actively investigated and improved, even though it has demonstrated exceptional effectiveness in treating other cancers, such as melanoma [4, 7]. Immunotherapy for CRC is currently only effective in people with MSI-H tumors and metastatic CRC. Immunomodulating drugs like levamisole have been studied in CRC treatment [4, 7, 31, 66]. Immune checkpoint inhibitors like pembrolizumab and nivolumab have demonstrated higher response rates and increased survival in these patients compared to conventional therapies [67]. Clinical trials have indicated that immune checkpoint inhibitors can effectively control disease progression, improving survival rates [11, 68]. Immunotherapy can be combined with other treatment options, like chemotherapy or targeted therapies, to enhance effectiveness. This synergistic approach has shown improved outcomes in clinical studies [69]. By identifying specific patient tumor characteristics, clinicians can determine if they are likely to respond to immunotherapy, enabling a more personalized and targeted approach (Fig. 1) [70].

### CAR-T-cell therapy and Adoptive cell therapy

Chimeric antigen receptor T (CAR-T) cell therapy, which involves engineering T cells from the patient’s immune system to target and destroy cancer cells, has been highly effective in treating certain leukemias, lymphomas, and other blood cancers [71]. CEA, mesothelin, guanylyl cyclase C, and epithelial cell adhesion molecule, amongst others, are the targets of CAR-T cell treatment for CRC. Leukapheresis, a therapy that collects T cells from the patient’s blood, is the first step in CAR-T cell therapy [72]. CARs are artificial receptors that can recognize specific proteins in cancer cells. In the field of targeting the appropriate antigens expressed explicitly in CRC cells, researchers are developing CARs [73]. The transformed T cells are grown and multiplied in the lab to create a large CAR-T cell population and reinfused into the recipient’s body. Clinical results on CAR-T cell treatment for CRC are few as it is currently in its initial stages [74]. Significant drawbacks of this treatment in CRC are high toxicity, relapses, and an impenetrable TME which also need to be considered [75, 76]. Adoptive cell therapy (ACT) enhances a patient’s immune cells to recognize better and eliminate cancer cells, showing promise in CRC treatment. This involves collecting and expanding T cells from a patient’s blood or tumor tissue, then reintroducing them to the patients to target cancer cells [76, 77]. Gene-engineered T cells and CAR-T cells are being explored to improve effectiveness and overcome immunosuppression in the TME. ACT is a highly personalized therapy that can lead to long-lasting effects and potentially a cure in some cases [76–78].

### Immune checkpoint inhibitors

Immune checkpoints include inhibitors of T cell activation like CTLA-4, PD-1, and PD-L1; promoters of T cell activation such as LAG3, OX40, and glucocorticoid-induced TNF receptor family-related protein; and those involved in T cell metabolism, like indoleamine 2,3-dioxygenase. Blocking suppressive checkpoints such as PD-1, PD-L1, and CTLA-4 has shown clear clinical benefits in MSIH/dMMR mCRC patients [79]. CTLA-4, found on T cells, inhibits T cell activation by outcompeting CD28 for binding to the costimulatory molecules CD80 and CD86 on antigen-presenting cells (APCs). T cells activated by tumor antigens presented by APCs circulate to locate matching antigens on tumor cells. An anti-tumor response is initiated when T cell receptors (TCRs) recognize these antigens on MHC molecules [80–82]. Inhibitors like pembrolizumab, nivolumab, and atezolizumab have effectively treated advanced CRC, especially in patients with MSI-H or dMMR tumors [59, 67]. These drugs block the interaction between immune checkpoint proteins PD-1 and PD-L1, enabling the immune system to recognize and destroy cancer cells. Identifying MSI-H or dMMR status in CRC has paved the way for ICIs as a promising therapeutic option [77, 80, 83]. Like PD-1/PD-L1 inhibitors, ICB restores the immune system’s capacity to identify and fight MSI-H/dMMR CRC cells by disrupting inhibitory signals [83]. Increased Tcell infiltration improves cytotoxic activity, longer survival, and lasting responses. MSI-H/dMMR CRC patients who have advanced on traditional therapies may use ICI therapy, which has acquired FDA clearance [84]. Inhibiting CTLA-4 and PD-1 simultaneously is the strategy currently used in some of the clinical studies. While PD-1 suppresses anti-tumor T cell responses later on, CTLA-4 prevents early T cell activation. In the phase II CheckMate-142 study, 119 previously treated patients with dMMR/MSI-H mCRC received nivolumab and ipilimumab, an anti-CTLA-4 IgG1 monoclonal antibody. The combination treatment showed promising results compared to nivolumab monotherapy: an objective response rate (ORR) of 55% vs. 31%, a 12-month progression-free survival (PFS) rate of 71% vs. 50%, and a 12-month overall survival rate of 85% vs. 73% [9]. Due to these promising outcomes, the FDA approved nivolumab + ipilimumab combination therapy for dMMR/MSI-H mCRC patients in July 2018 [9].

### Cancer vaccines

Cancer vaccines aim to induce a long-lasting and targeted immune response against the tumor to cause tumor shrinkage or control its growth [85]. Peptide-based vaccines are designed to deliver these antigens to the immune system to stimulate an immune response against the cancer [86]. Peptide-based vaccines, including the CEA and the MUC1 peptide vaccine, are effective against CRC [87]. A robust immune response is elicited towards the specific tumor antigen site by peptide-based vaccines, including chemical and biosynthetic formulations of expected or known specific tumor antigens [88]. A peptide-based vaccination can elicit a humoral immune response and create long-lasting immunological memory when paired with adjuvants [89].

**Fig. 2:**
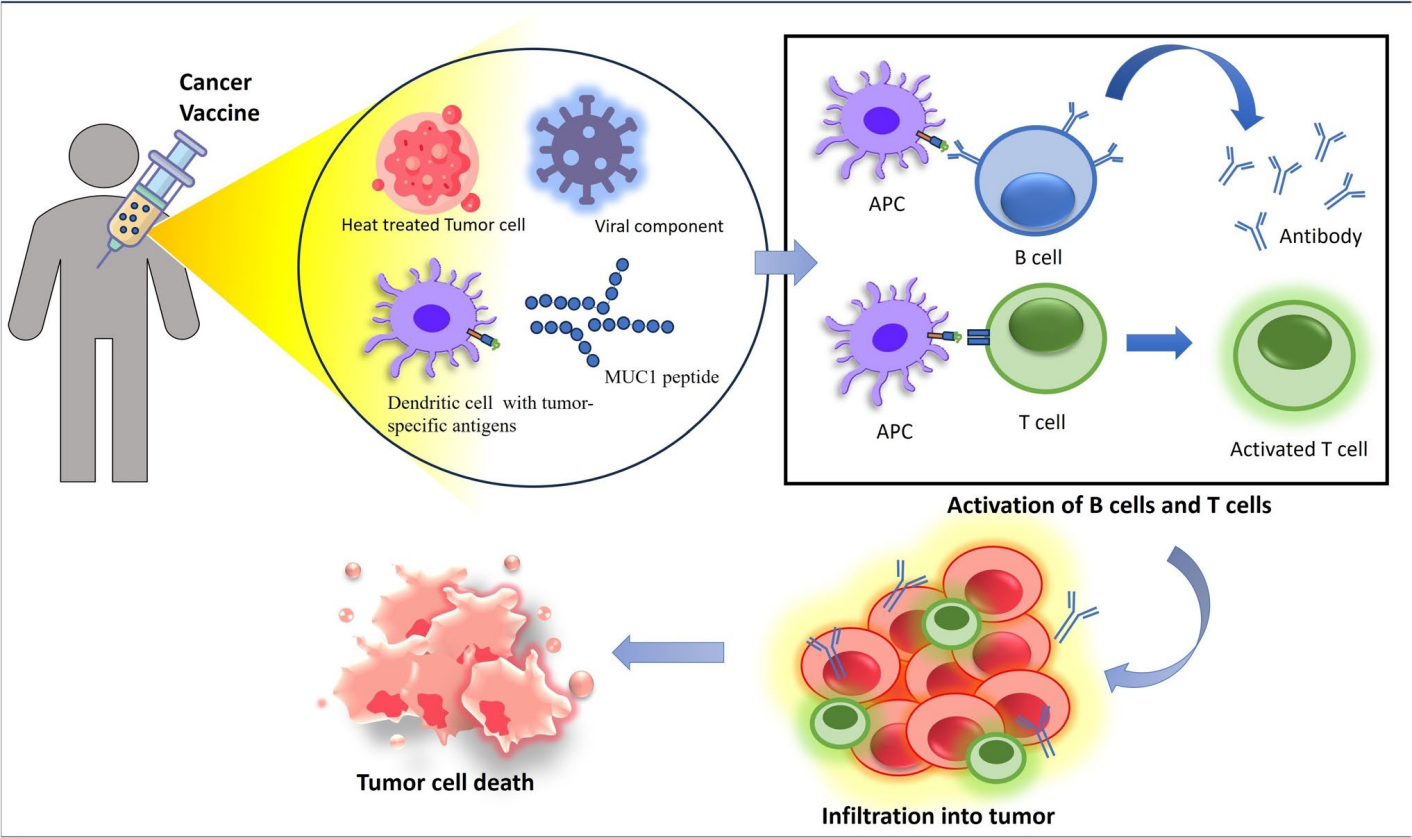
Diagram explaining the overview of the mechanism of action of cancer vaccines

The dendritic cell (DC) vaccine involves isolating a patient’s DC, loading them with tumor-specific antigens, and then reinfusing them into the individuals. These antigen-loaded DC can stimulate an immune response against the cancer cells [87, 89, 90]. Ongoing trials using DC vaccination to treat various human malignancies show promising results [91]. Complete tumor cells or fragments of tumor cells are used to elicit an immune response [92]. Viral vector-based vaccines use viruses that have been genetically modified to carry tumor-specific antigens. The viruses used in viral vector vaccinations have had their genomes altered to include one or more genes that encode for the desired antigens. The adenovirus-based vector is a commonly used viral vector for cancer vaccines [93, 94]. However, due to the multifaceted nature of CRC, it has become difficult to produce effective cancer vaccines. The inability of tumors to be recognized by the immune system, the existence of immunosuppressive TME, and tumor heterogeneity are a few of the reasons that restrict the effectiveness of vaccines (Fig. 2) [90–95].

## Biomarkers associated with immunotherapeutic response in CRC

Several biomarkers related to an immunologic response have been found in CRC. These biomarkers facilitate the selection of patients more likely to benefit from immunotherapy [96]. MSI-H or dMMR tumors have a mutation load and a higher rate of neoantigen generation. Compared to MSS tumors, patients with MSI-H or dMMR cancers showed significantly improved responses to immune checkpoint medicines such as anti-PD-1 antibodies [97]. It interacts with the PD-1 receptor on immune cells, resulting in immune evasion [98]. In CRC, the expression of PD-L1 is not an individual prognostic biomarker; instead, it is often used in conjunction with several other parameters [99]. High PD-L1 expression in CRC is linked to poor survival rates and is associated with lymph node metastasis and prognosis. Overexpression of PD-L1 in CRC is related to increased tumor mutation burden and microsatellite instability [100–103]. Improved clinical results have been shown when ICIs are administered to patients whose tumors have a high density of TIL, in particular, cytotoxic CD8 + T cells [100–105]. It has been shown that different immune gene expression profiles, such as IFN-γ signatures, may accurately predict a patient’s reaction to immunotherapy for CRC. These signs prove that immune pathways have been activated and that inflammation is present inside the TME [106, 107]. Although these biomarkers have been linked to immunotherapeutic response in CRC, their usefulness must be highlighted because prognostic biomarkers vary depending on the tumor’s molecular profile, the stage of the disease, and the individual patient’s immune landscape [106–108]. The MSI status is the best-established biomarker for immunotherapy response in the CRC [109]. Research is still being conducted to find other, more accurate indicators to improve patient selection and treatment results in immunotherapy for CRC [110].

## Combinational immunotherapy against CRC

**Fig. 3:**
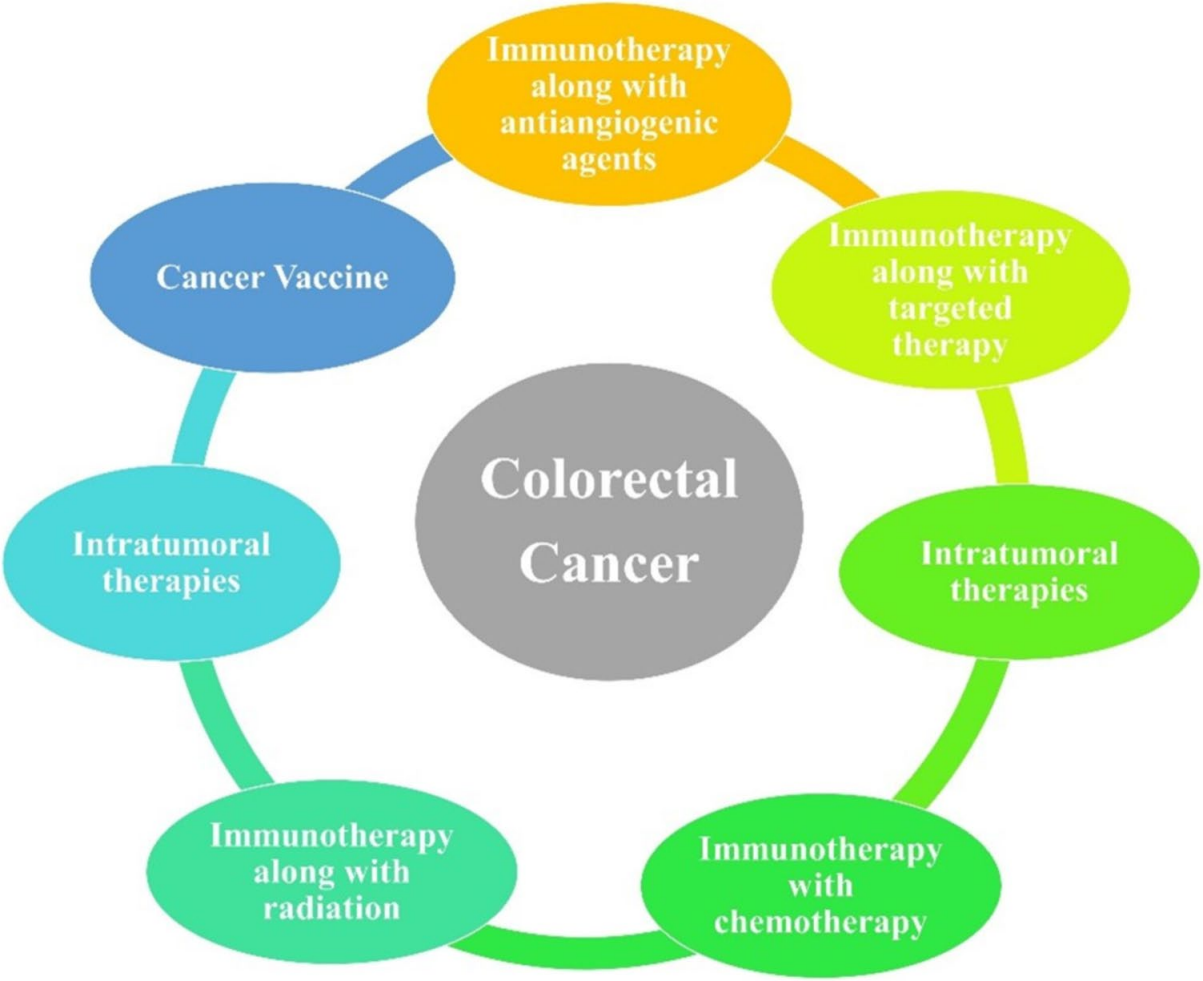
Diagram representing a few combinational approaches with immunotherapy against CRC

Due to insufficient TIL and restricted immunogenicity, most patients with CRC do not respond to ICIs. As a result, several therapeutic modalities have been studied to transform immunologically “cold” tumors into “hot” tumors by combining the anti-PD-1/PD-L1 antibodies with other immune-modulating therapies, including chemotherapy, radiotherapy, angiogenesis inhibitors, additional ICIs, and molecularly targeted medicines (Fig. 3) [46, 108, 110].

Synergistic effects may result from combinational immunotherapy, where different therapeutic strategies enhance overall efficacy [111]. Combinational immunotherapy may target the TME by regulating immune-resistant T cell populations, decreasing immunosuppression, and inducing an inflammatory and immune-active cancer microenvironment, ultimately improving the immune system’s ability to recognize and attack tumor cells [111, 112]. By combining medicines based on patient features or biomarkers, combinational immunotherapy allows for personalized treatment options [113] from which the CRC patients may be benefited [114].

Combinational immunotherapy in CRC enhances the immune response by combining immunotherapeutic drugs or immunotherapy with other treatments [115]. Anti-PD-1/PD-L1 and anti-CTLA-4 antibodies, along with targeted drugs like anti-EGFR and anti-VEGF, are being studied to boost T cell activation and anti-tumor immunity [116–118]. VEGF’s immunosuppressive effects can be countered by anti-angiogenic drugs, which have shown positive outcomes in clinical trials when paired with ICIs [119–122]. Combining ICIs with radiotherapy and chemotherapy, which induce immunogenic cell death (ICD), helps overcome resistance to immunotherapy. 5-Fluorouracil (5-FU) therapy increases TIL and the anti-tumor immune response by eradicating MDSC [121–124]. Combining chemotherapy regimens with ICIs has synergistic effects, enhancing immunotherapy effectiveness through mechanisms like tumor antigen release and immune response modulation [124, 125].

Combining chemotherapy and immunotherapy kills cancer cells more effectively and overcomes immune resistance [126]. ICIs or other immunomodulatory drugs are used alongside cancer vaccines to boost the immune response and kill cancer cells [127]. ACT may boost T cell activation and persistence in the cancer microenvironment when used with immunotherapy, such as checkpoint inhibitors. Localized radiation may boost tumor immunogenicity by killing immunogenic cells, releasing antigens, and altering the immune responses [128, 129].

Combining MAPK pathway-targeted therapy with immunotherapy is an area of investigation within CRC that shows promise in addressing immune-resistant pMMR/MSS CRC [129, 130]. Combined with immunotherapy, BRAF inhibitors, often used in treating BRAF-mutant CRC, have revealed a novel strategy to counteract immune resistance [130, 131]. MEK inhibitors, which target a downstream component of the MAPK pathway, have also demonstrated potential in combination with immunotherapy in destroying cancer cells [132, 133]. While these innovative combinations show promise, clinical trials are ongoing to determine their safety, efficacy, and optimal utilization [134]. Integrating MAPK pathway regulators with immunotherapy presents a novel prospect for advancing treatment options and improving outcomes for patients with immune-resistant pMMR/MSS CRC [129, 130, 135].

Furthermore, radiotherapy and immunotherapy work together to boost the immune system’s anti-tumor response, and combinational immunotherapy in CRC aims to overcome the limitations of single-agent immunotherapy and improve treatment results [136, 137]. However, the best combination approaches, treatment schedules, and patient selection criteria must be decided before treatment [138]. Clinical studies are assessing the safety and effectiveness of various combination methods, and further research is required to develop and verify the most effective combinational immunotherapy regimens for CRC treatment [139].

## Genomics and microbiome and their immunotherapy implications in CRC

CRC genomic instability varies based on DNA repair capability and is classified into four CMS subtypes: CMS 1 (immune), CMS 2 (canonical), CMS 3 (metabolic), and CMS 4 (mesenchymal) [140, 141]. MSI-high CRC, caused by mismatch repair deficiencies, leads to many mutations and a robust immune response, improving immunotherapy reactions [142–145]. Non-MSI CRCs show chromosomal instability with fewer rearrangements. Genetic abnormalities in *APC*, *TP53*, and *KRAS* alter cytokine production and immune cell recruitment [146–148]. Epigenetic changes, like DNA methylation, also plays a role in CRC development and can be targeted for treatment. Understanding these genetic and epigenetic factors is crucial for effective immunotherapy [149, 150]. The importance of the microbiome in the development of CRC is becoming more widely recognized, especially in its role in regulating the immune system in the colon [96, 151–153]. Recognizing the vital role of tissue-resident T lymphocytes and macrophage activation in immunotherapy, along with the microbiome’s influence on chemotherapy response, there is increasing interest in the interplay between the microbiome and cancer immunotherapy. The mouse models demonstrated that the inhibition of tumor growth and the effectiveness of anti-PD-L1 antibodies were linked to a high amount of *Bifidobacterium* [154–159]. Increased CD8 + T cell activity that explicitly targets tumors and the maturation of DC were shown to be associated with higher levels of *Bifidobacterium* [157, 160–163]. Studies found that specific microbial taxa with low abundances were associated with higher levels of CD3 + lymphocyte infiltration in CRC samples [161–163]. This correlation was observed alongside increased expression of CCR5 and CXCR3 chemokines, which play a role in the movement of T cells [160, 161]. Further, it was shown that bacteria with low abundance are crucial for enhancing the effectiveness of anti-PD-1 antibodies against mouse xenograft colon cancer models [162, 163]. This enhancement is achieved by increasing the infiltration of interferon (IFN)-γ + CD8 T cells into the tumor [162–164]. Studies have documented that patients with melanoma who were resistant to anti-PD-1 medication experienced either partial or total responses after receiving fecal transplants from individuals who responded positively to the treatment [165–168].

## Clinical applications and ongoing research

Current immunotherapy research in CRC aims to enhance treatment strategies and improve patient outcomes [169]. Immunotherapy shows significant promise for Lynch syndrome and MSI-H tumors, with drugs like nivolumab, ipilimumab, and pembrolizumab approved for mCRC [169, 170]. Trials evaluate combinations of atezolizumab with standard treatments for DNA mismatch repair deficiencies. Nivolumab and ipilimumab are tested with short-course radiation for MSI-H rectal cancer [171, 172]. For patients without Lynch syndrome, the research explored combining immunotherapy with other treatments like chemotherapy and targeted therapies, showing more benefits. Additional areas include CAR-T cell therapy, cancer vaccines, and oncolytic virus therapy, which are effective in treating CRC. Researchers are also investigating the action and pathways of immune modulators to boost the immune system. These advancements aim to refine therapeutic strategies and expand immunotherapy to various genetic profiles, improving patient care and outcomes, possibly in CRC treatment [173–176].

## Limitations and future perspectives

The TME, MSI status, and PD-L1 expression in CRC patients have been studied extensively. However, their efficacy in predicting treatment response is more complicated than other cancers and deserves further study. Lack of antigen presentation, changes in crucial immune signaling pathways, immune cell depletion, or alternate immune checkpoint pathways may be resistance mechanisms in cancer cell proliferation. Variability in patient immunotherapy responses complicates treatment outcome prediction [9]. Further, immunotherapy can cause organ-affecting immune-related adverse events (irAEs). Understanding risk variables such as patient characteristics, medication, and cancer type helps manage these events [11–15]. Another limitation is that the new immunotherapies are expensive and inaccessible to many individuals. Immunotherapy can have long-term impacts, although further research is needed [62, 145, 175].

Indicators like TIL and immune gene signatures must be studied beyond PD-L1 expression and MSI to improve direct therapeutic outcomes, and combining immunotherapy medications with chemotherapy or targeted therapies is required to improve CRC treatment. Multiple ICIs or immunomodulatory medicines may work synergistically to overcome immune resistance. Current research focuses on developing new immunotherapy drugs, such as bispecific antibodies or immune-stimulating nanoparticles, to improve CRC immune responses, which may be helpful in the future. The summary of the current review on the strategic approaches in CRC immunotherapy is mentioned in Table 1.

**Table 1:** Table representing the summary of the current review on the strategic approaches in CRC immunotherapy

Section	Key findings	Details
Introduction	Overview of immunotherapy	The document provides a comprehensive overview of recent advancements in immunotherapy for colorectal cancer, focusing on identifying effective therapeutic strategies and predictive biomarkers
Strategies	Tumor microenvironment: role of the immune system in cancer	Understanding the immune system’s role within the tumor microenvironment is crucial for identifying predictive biomarkers and developing innovative therapeutic strategies
Discussion	Potential for improved survival Enhancing immunotherapy efficacy Checkpoint inhibitors: success in MSI-H tumors	Combining immunotherapies, such as checkpoint inhibitors, may improve advanced colorectal cancer survival Using checkpoint inhibitors, targeted medicines, and conventional treatments, combinational immunotherapy is being studied to turn immunologically “cold” malignancies into “hot” ones, improving immune activation and treatment outcomes
Future prospects	Need for novel therapeutic approaches	Despite advancements, colorectal cancer prognosis remains poor, emphasizing the need for novel therapies that incorporate a deeper understanding of the tumor microenvironment and immune evasion mechanisms

## Discussion

CRC remains a leading cause of cancer-related deaths. While traditional treatments such as surgery, chemotherapy, and radiation therapy are commonly used, the prognosis for advanced-stage CRC is often poor [124]. Immunotherapy has shown promise, particularly for patients with dMMR or MSI-H tumors, who typically respond well to ICIs. However, these patients represent only about 15% of CRC cases, leaving the majority with pMMR and MSS tumors with fewer effective options [8]. Tumor heterogeneity complicates treatment, with factors such as low neoantigen presentation, impaired MHC-I antigen presentation, and an immunosuppressive TME involving cancer-associated fibroblasts and extracellular matrix [59]. Resistance to ICIs both primary and acquired and the lack of consensus on the optimal combination or sequencing with other therapies further complicate treatment strategies [9]. The gut microbiota also plays a crucial role in influencing ICIs efficacy, with dysbiosis contributing to resistance. Although combining radiotherapy with ICIs has the potential to improve responses, clinical success has been limited, highlighting the need for novel strategies and targets to expand the benefits of immunotherapy to a broader CRC population [169, 170].

Several strategies can be implemented to tackle the challenges of immunotherapy for CRC. First, enhancing biomarker identification is essential to predict better which patients will benefit from immunotherapy, especially for pMMR and MSS tumors where current biomarkers fall short [84]. Optimizing combination therapies through extensive clinical trials will help to identify the best combinations and sequencing with chemotherapy, targeted therapies, and radiotherapy for effective management of CRC [177]. Furthermore, the management of irAEs requires better monitoring and pre-emptive treatment strategies [170]. Reducing the high cost of novel immunotherapies and improving accessibility, particularly in resource-limited settings, is crucial to ensuring equitable treatment options for patients and maximizing the global impact of these advanced therapies in combating cancer and other diseases [8]. Research into novel targets and therapies for resistant CRC subtypes and conducting long-term studies to understand response durability and potential late effects are also necessary. Collaborative efforts among researchers, clinicians, and policymakers are vital to enhance the effectiveness and accessibility of immunotherapy for CRC treatment.

## Conclusion

Immunotherapy, remarkably ICIs targeting PD-1 or PD-L1, has significantly improved the treatment of advanced CRC, especially in patients with dMMR or MSI-H tumors. It offers better survival outcomes compared to chemotherapy and provides new options for those unresponsive to standard treatments. ICIs show higher response rates and prolonged disease control in MSI-H or dMMR CRC patients. Combining immunotherapy with chemotherapy or targeted therapies enhances effectiveness. Identifying predictive biomarkers, such as MSI or PD-L1 expression, also allows room for more personalized treatment. In conclusion, while immunotherapy has revolutionized the treatment landscape for specific CRC subtypes, continued innovation and collaboration are necessary to overcome existing limitations and bring these promising treatments to a broader array of patients. This article reviews relevant literature to help clinicians and researchers improve CRC survival rates through immunotherapy.

## Data Availability

No datasets were generated or analyzed during the current study.
